# Temporal Artery versus Bladder Thermometry during Adult Medical-Surgical Intensive Care Monitoring: An Observational Study

**DOI:** 10.1186/1471-2253-10-13

**Published:** 2010-08-12

**Authors:** Henry T Stelfox, Sharon E Straus, William A Ghali, John Conly, Kevin Laupland, Adriane Lewin

**Affiliations:** 1 Department of Critical Care Medicine, University of Calgary, Calgary, Canada; 2 Department of Medicine, University of Calgary, Calgary, Canada; 3 Department of Community Health Sciences, University of Calgary, Calgary, Canada; 4 Department of Medicine, Saint Michael's Hospital, University of Toronto, Toronto, Canada; 5 Department of Pathology and Laboratory Medicine, University of Calgary, Calgary, Canada; 6 Department of Microbiology and Infectious Diseases, University of Calgary, Calgary, Canada

## Abstract

**Background:**

We sought to evaluate agreement between a new and widely implemented method of temperature measurement in critical care, temporal artery thermometry and an established method of core temperature measurement, bladder thermometry as performed in clinical practice.

**Methods:**

Temperatures were simultaneously recorded hourly (n = 736 observations) using both devices as part of routine clinical monitoring in 14 critically ill adult patients with temperatures ranging ≥1°C prior to consent.

**Results:**

The mean difference between temporal artery and bladder temperatures measured was -0.44°C (95% confidence interval, -0.47°C to -0.41°C), with temporal artery readings lower than bladder temperatures. Agreement between the two devices was greatest for normothermia (36.0°C to < 38.3°C) (mean difference -0.35°C [95% confidence interval, -0.37°C to -0.33°C]). The temporal artery thermometer recorded higher temperatures during hypothermia (< 36°C) (mean difference 0.66°C [95% confidence interval, 0.53°C to 0.79°C]) and lower temperatures during hyperthermia (≥38.3°C) (mean difference -0.90°C [95% confidence interval, -0.99°C to -0.81°C]). The sensitivity for detecting fever (core temperature ≥38.3°C) using the temporal artery thermometer was 0.26 (95% confidence interval, 0.20 to 0.33), and the specificity was 0.99 (95% confidence interval, 0.98 to 0.99). The positive likelihood ratio for fever was 24.6 (95% confidence interval, 10.7 to 56.8); the negative likelihood ratio was 0.75 (95% confidence interval, 0.68 to 0.82).

**Conclusions:**

Temporal artery thermometry produces somewhat surprising disagreement with an established method of core temperature measurement and should not to be used in situations where body temperature needs to be measured with accuracy.

## Background

Accurate and precise temperature measurement is an important part of the clinical examination because an abnormal temperature reading can alter clinical management and patient prognosis[1]. The ideal system for measuring temperature should be minimally invasive and provide rapid results in a reliable, accurate and safe manner to support clinical decisions. However, questions still arise about the optimal method for temperature measurement in critically ill patients.

Most authorities including the American College of Critical Care Medicine and the Infectious Diseases Society of America consider temperature to be most accurately measured by intravascular, esophageal or bladder thermometry[2]. However, these devices are semi-invasive and may not be appropriate for use in all patients. Rectal thermometers are an alternative, but have limitations including patient perception that these are unpleasant and intrusive, access to the rectum may be limited by patient position, and patient movement may dislodge thermometers[2]. In addition, rectal thermometry has been associated with delays in recording temperature changes[3,4], and has been implicated in the spread of enteric pathogens[5]. Oral temperature measurement is safe, convenient, and familiar for alert and cooperative patients. However, in critically ill patients, oral temperatures are often not practical due to intubation or inability of the patient to cooperate. Furthermore, hyperventilation or ingested substances may affect oral temperature measurement[6,7]. The tympanic membrane temperature is believed to reflect the temperature of the hypothalamus and thus the core body temperature[8]. Infrared ear thermometry detects radiant energy from the tympanic membrane and ear canal through an otoscopic probe. However, multiple studies have shown consistently poor agreement between infrared tympanic membrane and rectal thermometers[9].

Infrared technology has been adapted to noninvasive temporal artery thermometry. Because the temporal artery has a high arterial perfusion rate that remains unchanged under most conditions, measurement of temperature via skin areas perfused by the temporal artery provides an easy, noninvasive estimate of core temperature[10]. This has resulted in wide adoption of the technology not only on general hospital wards, but also in intensive care units (ICU). However, professional society guidelines for measuring patient temperature in the ICU have recommended against using temporal artery thermometry because environmental temperature and sweating have been associated with unreliable temperature measurements[2,11]. Given the widespread clinical application of this new technology, we performed a prospective evaluation of the agreement in temperature assessment between a temporal artery thermometer and an established method of core temperature measurement, bladder thermometry as performed in clinical practice[12].

## Methods

### Study Subjects

We enrolled patients who were admitted to the medical-surgical ICU at Foothills Medical Centre between July 1, 2008 and November 28, 2008. We included patients who were 18 years of age or older, expected to stay in the ICU more than 24 hours and whose temperature was documented (using a temporal artery thermometer) in the medical record to have varied by at least 1°C (minimum range) in the 72 hours prior to being approached for consent. We selected these inclusion criteria to increase our prospects for capturing patients with dynamic temperature measurements. Patients were excluded if they: 1) were unable to provide consent and had no family member who could be approached for consent; 2) had any contraindication to bladder catheter thermometer placement and; 3) were diagnosed with an anatomic abnormality (e.g. skin abrasion or laceration) that would affect temperature measurement over the skin areas perfused by the temporal artery. The Conjoint Health Research Ethics Board at the University of Calgary approved the study protocol, and informed consent was obtained from either the patient or the closest family member before enrollment in the study.

### Instruments

Temperatures were measured from the skin overlying the temporal artery and from within the bladder. The *TemporalScanner *(TAT-5000, Exergen Corp., Watertown, MA, USA) was used for the infrared detection of temporal artery temperatures. Temperature measurements were performed according to the manufacturer's instructions (yearly in service training for bedside nurses). The *TemporalScanner's *probe was placed flush on the center of the patient's forehead, activated, slid across the skin from the forehead to the hairline, lifted from the forehead to behind the ear lobe and the temperature recorded. We used bladder temperature as the reference standard based on evidence that bladder thermometers show excellent agreement with pulmonary artery catheter thermometers over a wide range of temperatures[13,14] regardless of urine flow[15]. Temperature was measured using a bladder thermometer incorporated as part of a 16 French urinary drainage catheter (Level 1^® ^Foley Catheter temperature Sensor, Smiths Group PLC., Rockland, USA). Registered nurses inserted the catheters into patients who consented to participate. Both manufacturers reported calibrating the temporal artery (reported accuracy ± 0.1°C in the range of 16 to 43°C) and bladder (reported accuracy ± 0.2°C in the range of 5 to 45°C) thermometers prior to purchase. The clinical engineering department at the medical centre calibrated the temporal artery thermometers following purchase using the manufacturer's specifications.

### Procedure

Temperature recordings were obtained from the temporal artery and bladder as part of routine clinical monitoring performed by registered bedside nurses[12]. We ordered temperature measurements to be performed hourly and in a rapid, sequential manner during the study period. Data were recorded directly into the ICU's information system, TRACER, and time stamped. Temporal artery temperature measurements were manually entered into TRACER at the time of recording by the bedside nurse which is part of their usual care process. Bladder temperature measurements were continually displayed on the bedside monitor and validated (accepted by the system) by nursing staff. For the purposes of this study, bladder temperature measurements were designated as the core temperature. Fever was defined as a core temperature of greater than or equal to 38.3°C[2] based on guidelines jointly published by the American College of Critical Care Medicine and the Infectious Disease Society of America. Hypothermia was defined as a core temperature of less than 36.0°C[16].

### Statistical Analysis

The primary outcome was the agreement in temperature reading between the temporal artery thermometer and the bladder thermometer, with the latter considered as a reference standard. The secondary outcome was the accuracy (defined as the sensitivity and specificity) of the temporal artery thermometer to detect fever and hypothermia. Patient characteristics and temperature measurements were reported as means or medians for continuous variables, and as proportions for categorical variables. Temperature measurement agreement between methods was analyzed using the procedures described by Bland and Altman[17]. Limits of agreement were clinically (± 0.5°C) defined. Mean differences between temperatures from each method were calculated along with the standard deviations of the differences. The standard deviations were calculated using a one-way repeated measures analysis of variance with subject as the classification variable. To determine how similar temperature readings from the temporal artery thermometer were to the bladder thermometer in detecting fever, receiver operating characteristic analyses of sensitivity and specificity were performed and the area under the curve calculated with 95% confidence intervals. Statistical analysis was performed using R, version 2.9 (R Foundation for Statistical Computing, Vienna, Austria).

A preliminary analysis of data on two patients revealed a mean absolute difference between devices in temperature measured of 0.4°C with a standard deviation of 0.4°C. We therefore estimated that 14 patients with a minimum of 12 measurements would be needed to have 90% power to detect a conservative difference in temperatures measured of 0.3°C.

## Results

Table 1 summarizes the 14 patients enrolled in the study, 5 of whom were males and 9 females. The patients' ages ranged from 19 to 74 years (mean 51 ± 18). The diagnoses included trauma (n = 5), sepsis (n = 3), neurological disorders (n = 3) and other medical problems (n = 3). Patients were intubated (n = 14) and severely ill with a mean APACHE II score of 24 (± 5). During the study period, the majority of patients (9/14) received at least one temperature modifying intervention such as acetaminophen.

**Table 1:** Patient characteristics and thermometer readings.

Study Patients (n = 14)	
Baseline Characteristics	
Age (SD), yr	51 (18)
Sex (male/female)	5/9
Diagnosis, No (%)	
Major trauma	5 (36)
Sepsis	3 (21)
Neurological	3 (21)
Other	3 (21)
Surgery, No (%)	8 (57)
Intubated, No (%)	14 (100)
Vasopressor, No (%)	4 (29)
APACHE II Score (SD)	24 (5)
TISS Score (SD)	44 (9)
Temperature Modifying Interventions during Study	
Acetaminophen, No (%)	8 (57)
NSAID, No (%)	0 (0)
Hot/cool compresses, No (%)	2 (14)
Ice packs, No (%)	1 (7)
Heating/cooling blanket, No (%)	2 (14)
Heating/cooling catheter, No (%)	0 (0)
Heating/cooling vest, No (%)	0 (0)
**Thermometer Readings (n = 736 readings)**	
Temperature range,°C	
Temporal artery	35.7 - 39.4
Bladder	35.4 - 40.0
Mean (SD),°C	
Temporal artery	37.2 (0.69)
Bladder	37.7 (0.80)
Median (IQR),°C	
Temporal artery	37.2 (36.6 - 37.7)
Bladder	37.7 (37.2 - 38.2)
Hyperthermia [≥ 38.3°C], No (%)^†^	
Temporal artery	49 (7)
Bladder	166 (23)
Normothermia [36°C to < 38.3°C], No (%)	
Temporal artery	676 (92)
Bladder	545 (74)
Hypothermia [< 36.0°C], No (%)^‡^	
Temporal artery	11 (1)
Bladder	25 (3)

Abbreviations: NSAID; non-steroidal anti-inflammatory medication

† Hyperthermia was documented in 7 patients by temporal artery thermometer and 9 patients by bladder thermometer.

‡Hypothermia was documented in 3 patients by temporal artery thermometer and 2 patients by bladder thermometer.

A total of 760 temporal artery thermometer observations and 1,066 bladder thermometer observations were recorded from these 14 patients. Of these, 736 temporal artery and bladder temperature observations were recorded within one minute of each other and used for analysis. Table 1 summarizes the temperatures measured using the temporal artery and bladder thermometers. The mean difference between temporal artery and bladder temperatures was -0.44°C (95% confidence interval [CI], -0.47°C to -0.41°C), with temporal artery readings lower than bladder temperatures.

**Figure 1:**
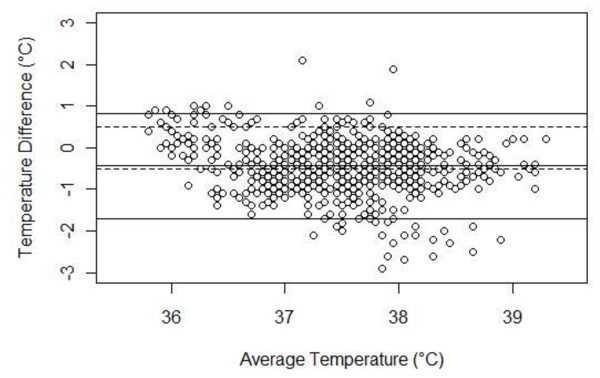
**Bland-Altman plot comparison of the difference between paired temporal artery and bladder (core) temperature measurements**. The middle solid horizontal line represents the mean difference in the two measurements, and the other solid horizontal lines demarcate 95% of observations. The two dashed horizontal lines represent a tolerance of ± 0.5°C.

Figure 1 summarizes the difference in temperature readings between the temporal artery and bladder. Agreement between the two devices was greatest for normothermia (36.0°C to < 38.3°C) (mean difference -0.35°C [95% CI, -0.37°C to -0.33°C]). The temporal artery thermometer recorded higher temperatures during hypothermia (< 36°C) (mean difference 0.66°C [95% CI, 0.53°C to 0.79°C]) and lower temperatures during hyperthermia (≥38.3°C) (mean difference -0.90°C [95% CI, -0.99°C to -0.81°C]).

**Figure 2:**
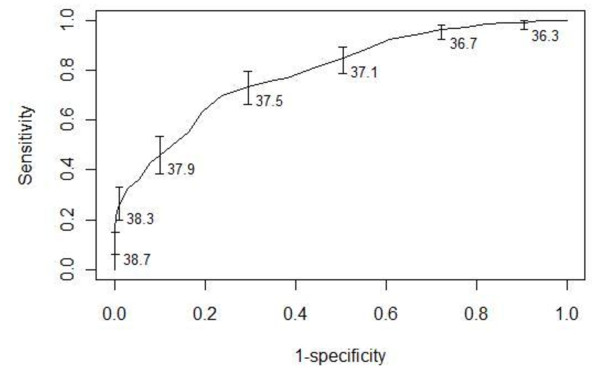
**Receiver operating characteristic curve for temporal artery thermometer in comparison with core temperature measurement by bladder thermometry**. Error bars represent 95% confidence intervals for the temperature thresholds presented in degrees Celsius.

Table 2 summarizes the operating characteristics of the temporal artery thermometer compared to the bladder thermometer for diagnosing fever. The temporal artery thermometer demonstrated a low sensitivity, but good specificity for diagnosing fever. Figure 2 summarizes the receiver operating characteristic curve of the temporal artery thermometer for diagnosing fever (area under the curve 0.80; 95% CI, 0.76 to 0.83). The accuracy of the temporal artery thermometer for diagnosing hypothermia was not assessed due to insufficient measurements of hypothermia (25/736, 3.4%).

**Table 2:** Sensitivity, specificity, positive likelihood ratio and negative likelihood ratio of the temporal artery thermometer compared with bladder thermometry (core) using 38.3°C as the cut-point for defining fever.

N = 736	Operating Characteristics (95% CI)
Sensitivity	0.26 (0.20 - 0.33)
Specificity	0.99 (0.98 - 0.99)
Positive Likelihood Ratio	24.6 (10.7 - 56.8)
Negative Likelihood Ratio	0.75 (0.68 - 0.82)

## Discussion

We performed a prospective evaluation of the agreement in temperature assessment between a widely adopted new methodology, temporal artery thermometry and an established core measurement methodology, bladder thermometry as performed in clinical practice[12]. The temporal artery thermometer closely agreed with the bladder thermometer for normothermic (36°C to < 38.3°C) temperatures. However, there was less agreement for temperatures below 36°C and temperatures greater than or equal to 38.3°C. The temporal artery thermometer had low sensitivity, but good specificity for diagnosing fever.

Our data support previous studies that have noted a lack of agreement between temporal artery thermometry measurements and standard core temperature measurement sites: bladder, pulmonary artery, rectum, axillary and tympanic membrane[18-23]. Temporal artery thermometers appear superior to axillary and tympanic membrane infrared thermometers, but inferior to bladder, pulmonary artery and rectal thermometers[18-23]. Suleman et al. compared a temporal artery thermometer with pulmonary artery and bladder thermometers in patients post-cardiac surgery and with a temperature of at least 37.8°C[20]. The performance of the thermometer was poor with a sensitivity of 0%, but specificity of 100% for fever in their adult patients[20]. Conversely, Greenes and Fleisher determined that in a sample of infants evaluated in the emergency department, the temporal artery was accurate for those with a high rectal fever and recommended it as an alternative to tympanic membrane thermometry[21]. Schuh et al. found the temporal artery thermometer to be an effective screening instrument for fever in children under 24 months of age, but insufficiently accurate to replace rectal thermometry[22]. Hebbar et al. judged the temporal artery thermometer to demonstrate marked variability compared to rectal temperature measurements in critically ill pediatric patients and recommended against it replacing invasive core temperature measurements[19]. However, Myny et al. evaluated the temporal artery thermometer against a pulmonary artery thermometer in a sample of normothermic critically ill adult patients and judged it to have acceptable reliability and accuracy[24]. Similarly, Lawson et al. demonstrated reasonable agreement between pulmonary artery catheter thermometers and both temporal artery and oral thermometers, but poor agreement with axillary and tympanic thermometers[23]. Kimberger et al. evaluated a temporal artery thermometer peri-operatively in a neurosurgical operating room and post-operative ICU and concluded that it was not an adequate substitute for core temperature monitoring[18].

Our study adds to the existing literature by providing the single largest clinical evaluation (736 paired observations) of a temporal artery thermometer in critically ill adult patients with a wide temperature range (35.4 to 40.0°C) in a medical-surgical ICU. In the ICU, temperature measurement is an essential component of patient assessment and management decisions. Our data highlight that although the temporal artery thermometer is convenient to use, minimally invasive and provides rapid results it agrees insufficiently with an established core temperature measurement methodology to be clinically useful in patients where careful temperature monitoring is important (e.g. sepsis surveillance, neurological injury, active heating or cooling of patients). This is most clearly illustrated by the small mean difference between the temporal artery and bladder thermometers for normothermic temperatures, but much larger mean differences for hypothermic and hyperthermic temperatures. This would give the instrument low sensitivity and the inability to rule out either hypothermia or fever. Conversely, our data demonstrate that the high specificity of the instrument indicates that if a fever is identified with a temporal artery thermometer then it is likely a true positive. Our results are perhaps not surprising given that skin temperature measurement can be potentially affected by patient (regional perfusion, diaphoresis, vasoactive medications), instrument (dirt or oil on temporal artery thermometer lens) and environmental (external heating or cooling devices such as fans) factors[2,11,25].

The results of our study need to be interpreted within the context of its limitations. First, although a wide range of temperatures was recorded in our study, there were few recordings of hypothermia. Thus, statements about the performance of temporal artery thermometry under conditions of hypothermia are limited. Second, we employed a bladder thermometer as our reference standard. Although there is no universally accepted reference standard for measuring core temperature, some authorities consider a pulmonary artery thermometer to be the standard against which other devices should be compared[2]. However, pulmonary artery catheters are invasive and employed infrequently in clinical practice[26]. Conversely, bladder thermometers are less invasive and provide continuous readings that are essentially identical to intravascular thermometers over a wide range of temperatures regardless of urine flow[13-15]. Third, we did not independently validate thermometer calibration or monitor nursing compliance with the recommended procedure for using the temporal artery thermometer which may have contributed to suboptimal performance. As such our study reflects the realities of a clinical as opposed to a laboratory evaluation of the temporal artery thermometer[12].

## Conclusions

In summary temporal artery thermometer measurements agreed with bladder thermometer measurements over normothermic temperature ranges, but demonstrated limited agreement for temperatures below 36°C and temperatures greater than or equal to 38.3°C. Temporal artery thermometry produces somewhat surprising disagreement with an established method of core temperature measurement and should not be used in situations where body temperature needs to be measured with accuracy.

## Abbreviations

ICU: intensive care unit; CI: confidence interval.

## Competing interests

Funding sources had no role in the design, conduct, or reporting of this study and we are unaware of any conflicts of interest. Dr. Stelfox had full access to all of the data in the study and takes responsibility for the integrity of the data and the accuracy of the data analysis.

## Authors' contributions

HTS designed the study, collected, analyzed and interpreted the data, and drafted and revised the manuscript. SES designed the study, interpreted the data and revised the manuscript. WAG analyzed and interpreted the data and revised the manuscript. JC designed the study, interpreted the data and revised the manuscript. KL designed the study, interpreted the data and revised the manuscript. AL analyzed and interpreted the data and drafted and revised the manuscript. All authors read and approved the final manuscript.

## Pre-publication history

The pre-publication history for this paper can be accessed here:

http://www.biomedcentral.com/1471-2253/10/13/prepub
